# Prognostic Value of the Right Ventricular-to-Left Ventricular Volume Ratio in Tricuspid Regurgitation

**DOI:** 10.1016/j.jacadv.2025.101922

**Published:** 2025-07-04

**Authors:** Robert S. Zhang, Pablo Villar-Calle, Lily Jin, Rachel Axman, Zachary Falk, Mahniz Reza, Annie Tsay, Giorgia Falco, Andre Cheng, Shmuel Chen, Jonathan W. Weinsaft, Jiwon Kim

**Affiliations:** a Division of Cardiology, Weill Cornell Medicine, New York, New York, USA; b Department of Medicine, Weill Cornell Medicine, New York, New York, USA

**Keywords:** magnetic resonance imaging, remodeling, right ventricle, tricuspid regurgitation

## Abstract

**Background:**

Tricuspid regurgitation (TR) is associated with right ventricular (RV) remodeling; however, conventional RV metrics may not fully reflect the interplay between the right and left ventricles.

**Objectives:**

The aim of the study was to examine the prognostic value of the right ventricular-to-left ventricular volume ratio (RV/LV_vol_) ratio in TR.

**Methods:**

A retrospective analysis was conducted on 949 patients with ≥moderate TR who underwent cardiac magnetic resonance imaging between 2005 and 2024. The RV/LV_vol_ ratio was assessed as a dichotomous variable (normal: <1.27, abnormal: ≥1.27) and by severity strata. Follow-up data, including all-cause mortality, were collected using the Social Security Death Index and electronic medical records.

**Results:**

Of the 949 patients, 43.6% had an abnormal RV/LV_vol_ ratio. Among 528 patients with a normal RV end-diastolic volume index, 178 (33.7%) had an abnormal RV/LV_vol_ ratio. Over a mean follow-up of 4.8 ± 4.5 years, 236 patients died. An abnormal RV/LV_vol_ ratio was independently associated with increased mortality after adjusting for covariates (adjusted HR: 1.47, 95% CI: 1.01 to 2.14, *P* = 0.043). Mortality risk increased with RV/LV_vol_ ratio severity, with severe ratios conferring the highest risk (adjusted HR: 2.20, 95% CI: 1.31-4.76, *P* = 0.045). The RV/LV_vol_ ratio provided significant incremental prognostic value over conventional RV indices, improving global chi-square from 24.7 (age/sex) to 47.1 with RV ejection fraction, 59.3 with RV end-diastolic volume index, and 68.3 with the addition of RV/LV_vol_ ratio (*P* = 0.005).

**Conclusions:**

The RV/LV_vol_ ratio is a strong predictor of mortality in advanced TR, capturing ventricular remodeling not identified by conventional metrics.

Tricuspid regurgitation (TR) is a prevalent and clinically challenging condition, with moderate to severe TR affecting 1.6 million people in the United States.^1^ Despite high prevalence, TR often remains undertreated until advanced stages, when irreversible right ventricular (RV) remodeling has already occurred. Progressive RV dilation and systolic dysfunction create a cycle of progressive annular dilation and regurgitation, further compromising RV function and increasing the risk of adverse outcomes, including heart failure and death.^1^ Given the significant morbidity and mortality associated with TR, early identification of high-risk patients is essential to improving outcomes and guiding timely intervention.

Historically, the clinical management of TR has focused on optimizing volume status with diuretics, which do not provide mortality benefit, while surgical intervention has been limited by high perioperative risk.^2^ The recent emergence of transcatheter tricuspid valve intervention (TVI) offers a promising therapeutic solution, potentially expanding treatment options for patients who were previously considered ineligible for intervention.^3^ However, the optimal timing of TVI remains a topic of ongoing debate. Current guidelines often emphasize RV dilatation as a key criterion for intervention, but there is no consensus on ideal thresholds for RV remodeling to guide decision-making.^2^

Cardiac magnetic resonance (CMR) imaging has emerged as the reference standard for RV quantification, providing accurate and reproducible volumetric analysis.^3^ However, even with CMR, traditional parameters such as indexed RV end-diastolic volume index (RVEDVi) may underestimate the degree of RV dilation due to their broad range of normal values and failure to account for individual variations in heart size.^4^ Clinically, there are instances where the RV may appear larger than the left ventricle (LV), yet the RVEDVi remains within normal limits. The RV/LV volume (RV/LV_vol_) ratio has been proposed as an alternative metric and has been validated as a prognostic marker in pulmonary hypertension, pulmonary embolism, and pulmonic regurgitation.^4^, ^5^, ^6^, ^7^ This ratio has the potential to serve as a more comprehensive marker of TR severity, as increasing TR severity would plausibly correlate with a higher RV/LV_vol_ ratio. Given the limitations of conventional RV indices and the emerging evidence supporting the prognostic value of the RV/LV_vol_ ratio, we hypothesized that the RV/LV_vol_ ratio, as assessed by CMR, would serve as a robust predictor of mortality in patients with advanced TR, providing incremental prognostic value beyond conventional indices.

## Methods

### Population

The study population included consecutive patients who underwent CMR at Weill Cornell Medicine, New York-Presbyterian Hospital between July 2005 and June 2024 and had at least moderate TR on CMR. Advanced TR was evaluated by 2 level 3 CMR readers using a qualitative, multiparametric, integrative approach. This assessment incorporated right atrial enlargement, lack of leaflet coaptation, vena contracta >3 mm, signal void area occupying >25% of the right atrium, RV enlargement, tricuspid annular dilation, and quantitative measures such as a regurgitant volume ≥30 mL or a regurgitant fraction ≥30%.^8^, ^9^, ^10^, ^11^ For patients with multiple CMR exams, the initial study where advanced TR was diagnosed was utilized for analysis. Patients with prior tricuspid valve repair or replacement and congenital or primary tricuspid pathologies (prolapse, rheumatic, and leaflet perforation) were excluded.

A hierarchical classification system was used to determine the predominant etiology of TR, categorizing it as resulting from left-sided heart disease (>moderate aortic or mitral valve disease, left ventricular ejection fraction [LVEF] <50%), pulmonary hypertension (documented pulmonary hypertension [PH] or pulmonary artery systolic pressure ≥50 mm Hg), atrial fibrillation, or isolated causes.

Comprehensive clinical and demographic data were collected via medical record review. All-cause mortality was assessed using the Social Security Death Index, supplemented by local electronic medical records.

The WCM Institutional Review Board provided approval for this study, including a waiver of informed consent for the use of pre-existing imaging and clinical data as analyzed for research purposes. Data, analytic methods, and study materials can be made available for result reproduction or procedure replication upon reasonable request.

### Image acquisition

CMR was performed using 1.5-T or 3.0-T scanners. Cine-CMR imaging was conducted using a steady-state free precession pulse sequence in contiguous LV short-axis and long-axis orientations (2-, 3-, and 4-chamber views). Gadolinium-based contrast agents (total dose 0.15-0.20 mmol/kg) were administered, and late gadolinium enhancement (LGE)-CMR imaging was performed approximately 10 minutes after contrast injection using orientations matched to cine-CMR.

### Image analysis

LV and RV chamber size and systolic function were quantified volumetrically on cine-CMR. End-diastolic and end-systolic chamber volumes were quantified via endocardial border planimetry of contiguous short axis images, with results used to calculate ejection fraction (EF) for each respective chamber. Prior studies by our group have documented high reproducibility for both LV and RV quantitative analyses using methods employed in this study.^12^^,^^13^ Thresholds for RVEDVi were >109 mL/m^2^ for men and 97 mL/m^2^ for women, in accordance with published reference ranges.^14^ LGE was evaluated using an established semiquantitative method.^15^ For the RV/LV_vol_ ratio, a uniform cutoff (≥1.27) was applied and was further tested using gradation thresholds (mild: 1.27 to <1.69, moderate: 1.69 to <2.29, severe: ≥2.29) employed in previous studies.^4^^,^^5^^,^^16^

To evaluate the reproducibility of TR severity, intraobserver and interobserver reproducibility analyses were conducted. For intraobserver reproducibility, 1 observer repeated the measurements twice, with a 1-week interval between assessments. For interobserver reproducibility, a second independent observer performed the measurements on the same parameters. Both analyses were conducted on a randomly selected subset of 20 patients (Supplemental Table 1). All observers were blinded to patient clinical data to maintain objectivity.

### Statistical analysis

Continuous data were compared using Student's *t*-test (2 groups), while analysis of variance was used to compare >2 groups. Categorical data were compared using the chi-squared test. To assess the relationship between the RV/LV_vol_ ratio and all-cause mortality, Cox proportional hazards (CPH) models were constructed with the RV/LV_vol_ ratio entered as a dichotomous variable and adjusted for age, sex, RVEDVi (per 10 mL/m^2^ increase), right ventricular ejection fraction (RVEF, per 10% increase), left ventricular end-diastolic volume index (LVEDVi, per 10 mL/m^2^ increase), LVEF (per 10% increase), and LGE. A similar analysis was conducted using RV/LV_vol_ ratio severity strata. The proportional hazard assumption for each model was tested with Schoenfeld residuals.^17^ The linearity assumption for continuous variables in the CPH model was assessed using restricted cubic splines. For each continuous covariate, spline terms were generated, and model fit was compared to the corresponding linear model using likelihood ratio tests. A two-sided *P* value <0.05 was considered evidence of a nonlinear relationship. To assess multicollinearity among the CMR variables included in the multivariable model, we calculated variance inflation factors using a linear regression with the same covariates.

Subsequently, we performed a forward stepwise CPH model to evaluate the incremental prognostic value of RV/LV_vol_ ratio over baseline demographics and other RV parameters. In the model, age was entered first, followed by RVEF (per 10% increase), then RVEDVi (per 10 mL/m^2^ increase), LVEF (per 10% increase), and finally the RV/LV_vol_ ratio. For each model, the incremental predictive power was assessed according to increments in the global chi-square values by comparing the likelihood ratio of full and reduced models. Results are presented using the adjusted HR (aHR) with 95% CI.

A Kaplan-Meier curve was generated for the 2 groups (normal: <1.27, abnormal: ≥1.27) and based on prior established severity cutoffs (mild: 1.27 to <1.69, moderate: 1.69 to <2.29, severe: ≥2.29).^4^^,^^5^ The log-rank test was used to compare all-cause mortality between the groups. All tests were considered significant at a two-sided alpha level <0.05. All analyses were performed using Stata software (StataCorp 18 LP).

## Results

### Baseline characteristics

The study cohort included 949 patients with advanced functional TR who underwent CMR. Of the 949 patients, TR etiology was attributed to left-sided heart disease in 460 patients (48.5%), pulmonary hypertension in 341 patients (35.9%), atrial fibrillation in 103 patients (10.9%), and isolated TR in the remaining 45 patients (4.7%). A small subset of patients (1.8%, 17/949) had ≥moderate pulmonic regurgitation. A total of 453 of 949 (48%) patients had quantitative measurements for advanced TR.

Patients with severe TR demonstrated a higher RV/LV_vol_ ratio (1.79 ± 0.83) compared to those with moderate TR (1.28 ± 0.80; *P* < 0.001). Similarly, the prevalence of an abnormal RV/LV_vol_ ratio (≥1.27) was nearly double in the severe TR cohort (67.9% vs 38.1%, *P* < 0.001). Among the study cohort, 414 (43.6%) had an abnormal RV/LV_vol_ ratio (Central Illustration), categorized as mildly abnormal in 234 patients (24.7%), moderately abnormal in 104 patients (10.9%), and severely abnormal in 76 patients (8.0%). Of the 528 patients with a normal RVEDVi, 178 (33.7%) had an abnormal RV/LV_vol_ ratio. Women were less likely to have an abnormal RVEDVi (39.5% vs 49.9%, *P* = 0.004). Notably, 18.4% of patients with a severely abnormal RV/LV_vol_ ratio had a normal RVEDVi based on current guideline-recommended cutoffs.

**Central Illustration:**
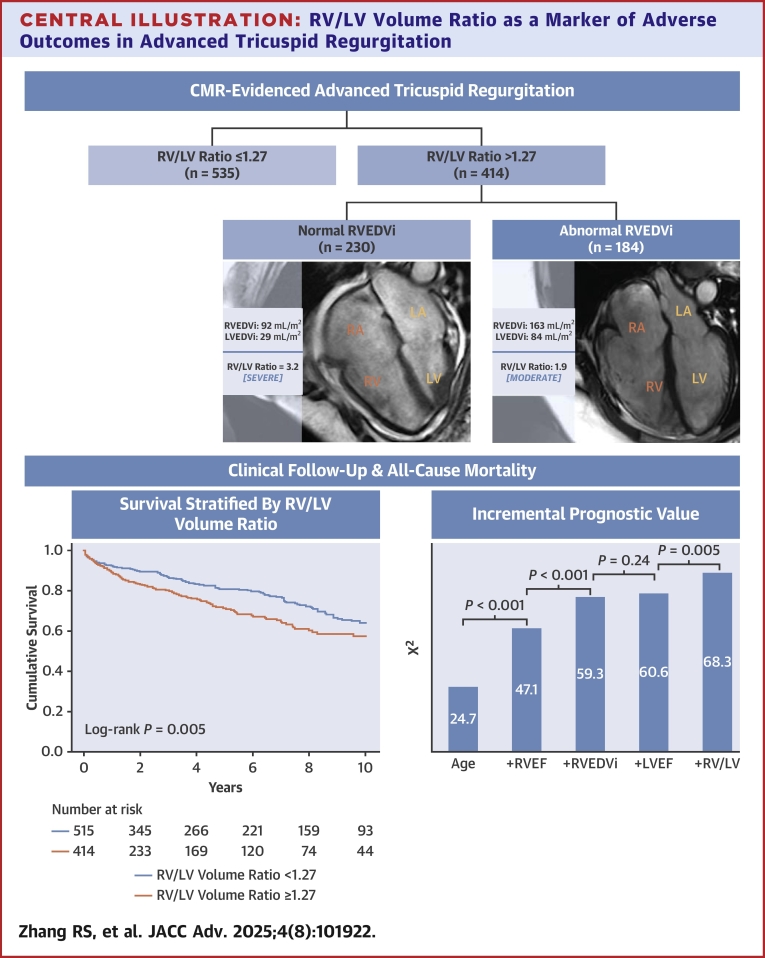
RV/LV Volume Ratio as a Marker of Adverse Outcomes in Advanced Tricuspid Regurgitation Proportion of patients with an abnormal RV/LV volume ratio and normal vs abnormal RVEDVi in CMR-evidenced advanced tricuspid regurgitation, and the impact of RV/LV volume ratio on all-cause mortality. CMR = cardiac magnetic resonance; LVEDVi = left ventricular end-diastolic volume index; other abbreviations as in Figure 3.

As shown in Table 1, patients with an RV/LV_vol_ ratio were less likely to be men (44% vs 56.8%, *P* < 0.001) and had smaller LV size as demonstrated by lower LVEDVi (65.1 ± 20.5 vs 101.8 ± 39.8 mL/m^2^, *P* < 0.001) and LV mass (60.6 ± 20.3 vs 77.0 ± 27.1 mL/m^2^, *P* < 0.001). They also had higher LVEF (58.8% ± 13.1% vs 42.6% ± 19.1%, *P* < 0.001), higher RVEDVi (119.1 ± 74.9 vs 96.1 ± 34.6 mL/m^2^, *P* < 0.001), and lower prevalence of LGE (2.1% ± 4.9% vs 6.7% ± 10.9%, *P* < 0.001).

**Table 1:** Baseline Characteristics and Cardiac Function Stratified by RV/LV Volume Ratio

	RV/LV_vol_ Ratio <1.27 (n = 535)	RV/LV_vol_ Ratio ≥1.27 (n = 414)	*P* Value
Age (y)	71.6 ± 15.1	70.8 ± 16.0	0.39
Male	304 (56.8%)	182 (44.0%)	**<0.001**
BSA (m^2^)	1.9 ± 0.3	1.8 ± 0.3	**0.01**
Cardiovascular risk factors			
Diabetes mellitus	127 (24.0%)	93 (22.7%)	0.65
Hypertension	332 (62.4%)	274 (66.5%)	0.19
Hyperlipidemia	269 (50.8%)	223 (54.3%)	0.29
Chronic kidney disease	82 (15.6%)	51 (12.5%)	0.18
Atrial fibrillation	198 (37.4%)	172 (41.8%)	0.17
Prior myocardial infarction	110 (20.6%)	74 (18.0%)	0.30
COPD	54 (10.2%)	56 (13.7%)	0.11
Pulmonary hypertension	287 (54.4%)	212 (51.5%)	0.38
Prior CVA/TIA	52 (9.9%)	50 (12.2%)	0.26
Cardiovascular medications			
Aspirin	231 (44.3%)	151 (36.8%)	**0.02**
Statin	230 (44.1%)	197 (48.0%)	0.24
Beta-blocker	293 (56.2%)	238 (58.0%)	0.58
ACE inhibitor/ARB	210 (40.3%)	153 (37.3%)	0.35
Aldosterone antagonist	102 (19.6%)	65 (15.9%)	0.14
Loop diuretic	210 (40.3%)	163 (39.8%)	0.86
SGLT-2 inhibitor	10 (1.9%)	14 (3.4%)	0.15
Sacubitril-valsartan	12 (2.3%)	16 (3.9%)	0.16
LV geometry/function			
LVEDVi (mL/m^2^)	101.8 ± 39.8	65.1 ± 20.5	**<0.001**
LV dilation^a^	216 (40.4%)	21 (5.1%)	**<0.001**
LVEDV, ml	192.5 ± 82.1	120.2 ± 44.8	**<0.001**
LV mass indexed (g/m^2^)	77.0 ± 27.1	60.6 ± 20.3	**<0.001**
LVEF (%)	42.6 ± 19.1	58.8 ± 13.1	**<0.001**
LGE	6.7 (10.9%)	2.1 (4.9%)	**<0.001**
RV geometry/function			
RVEDVi (mL/m^2^)	96.1 ± 34.6	119.1 ± 74.9	**<0.001**
RV dilation^b^	125 (23.4%)	184 (44.4%)	**<0.001**
RVEDV (mL)	181.7 ± 73.2	218.5 ± 135.9	**<0.001**
RVEF (%)	45.2 ± 13.6	45.3 ± 11.8	0.93
TR severity			
Moderate	475 (62.3%)	287 (37.7%)	**<0.001**
Severe	60 (32.1%)	127 (67.9%)	**<0.001**
Regurgitant volume (mL)^c^	29 ± 9.3	40 ± 18	**0.008**
Regurgitant fraction (%)^c^	46 ± 12	43 ± 11	0.16

Bold values indicate statistical significance (*P* < 0.05).

ACE inhibitor/ARB = angiotensin-converting enzyme inhibitor/angiotensin receptor blocker; BSA = body surface area; COPD = chronic obstructive pulmonary disease; CVA/TIA = cerebrovascular accident/transient ischemic attack; LGE = late gadolinium enhancement; LV = left ventricular; LVEDV = left ventricular end-diastolic volume; LVEDVi = left ventricular end-diastolic volume index; LVEF = left ventricular ejection fraction; RV = right ventricular; RVEDV = right ventricular end-diastolic volume; RVEDVi = right ventricular end-diastolic volume index; RVEF = right ventricular ejection fraction; RV/LV volume ratio = right ventricular to left ventricular volume ratio; SGLT-2 = sodium-glucose co-transporter-2; TR = tricuspid regurgitation.

a LVEDVi >108 mL/m^2^ for men; LVEDVi >96 mL/m^2^ for^14^ women.

b RVEDVi >109 mL/m^2^ for men; RVEDVi >97 mL/m^2^ for^14^ women.

c Available in 453 patients; mean ± SD; count (percentage).

Patients stratified by RV/LV_vol_ ratio severity demonstrated a stepwise progression in clinical and remodeling characteristics (Table 2). Those with a severely abnormal RV/LV_vol_ ratio (≥2.29) were younger (67.5 ± 15.4 vs 71.6 ± 15.1 years in the normal group, *P* = 0.001) and more likely to be female (68.4% vs 43.2%, *P* < 0.001). Body surface area also declined with increasing RV/LV_vol_ ratio severity (*P* = 0.038). Significant differences in ventricular remodeling were also observed across RV/LV_vol_ ratio severity groups. LVEDVi decreased progressively, from 101.8 ± 39.8 mL/m^2^ in the normal group to 51.1 ± 18.0 mL/m^2^ in the severe group (*P* < 0.001), and the prevalence of LV dilation decreased significantly from 40.4% to 1.3% (*P* < 0.001). In contrast, RVEDVi increased markedly, from 96.1 ± 34.6 mL/m^2^ in the normal group to 170.4 ± 151.6 mL/m^2^ in the severe group (*P* < 0.001), with RV dilation prevalence rising significantly from 23.4% to 81.6% (*P* < 0.001). RVEF declined progressively, reaching its lowest in the severe group (34.2% ± 10.4% vs 45.2% ± 13.6%, *P* < 0.001), further highlighting the robustness of RV/LV_vol_ ratio strata to stratify disease severity.

**Table 2:** Baseline Characteristics and Cardiac Function Stratified by RV/LV Ratio Categories

	Normal (n = 535) (RV/LV <1.27)	Mild (n = 234) (RV/LV ≥1.27-<1.69)	Moderate (n = 104) (RV/LV ≥1.69-<2.29)	Severe (n = 78) (RV/LV ≥2.29)	*P* Value
Age (y)	71.6 ± 15.1	71.8 ± 14.7	70.9 ± 18.8	67.5 ± 15.4	0.16
Male	304 (56.8%)	113 (48.3%)	45 (43.3%)	24 (31.6%)	**<0.001**
BSA (m^2^)	1.9 ± 0.3	1.9 ± 0.3	1.8 ± 0.3	1.8 ± 0.2	**0.038**
Cardiovascular risk factors					
Diabetes mellitus	127 (24.0%)	51 (22.0%)	25 (24.0%)	17 (23.0%)	0.94
Hypertension	332 (62.4%)	145 (62.5%)	72 (69.2%)	57 (75.0%)	0.11
Hyperlipidemia	269 (50.8%)	127 (54.5%)	56 (53.8%)	40 (54.1%)	0.76
Chronic kidney disease	82 (15.6%)	26 (11.2%)	11 (10.6%)	14 (19.2%)	0.16
Atrial fibrillation	198 (37.4%)	89 (38.2%)	51 (49.0%)	32 (43.2%)	0.14
Prior myocardial infarction	110 (20.6%)	50 (21.4%)	14 (13.5%)	10 (13.5%)	0.17
COPD	54 (10.2%)	38 (16.4%)	10 (9.6%)	8 (10.8%)	0.091
Pulmonary hypertension	287 (54.4%)	108 (46.4%)	60 (58.3%)	44 (57.9%)	0.090
Prior CVA/TIA	52 (9.9%)	28 (12.1%)	15 (14.4%)	7 (9.5%)	0.49
Cardiovascular medications					
Aspirin	231 (44.3%)	90 (38.6%)	39 (37.5%)	22 (30.1%)	0.072
Statin	230 (44.1%)	118 (50.6%)	48 (46.2%)	31 (42.5%)	0.38
Beta-blocker	293 (56.2%)	133 (57.1%)	62 (59.6%)	43 (58.9%)	0.91
ACE inhibitor/ARB	210 (40.3%)	87 (37.3%)	35 (33.7%)	31 (42.5%)	0.52
Aldosterone antagonist	102 (19.6%)	39 (16.7%)	14 (13.5%)	12 (16.4%)	0.44
Loop diuretic	210 (40.3%)	87 (37.3%)	47 (45.2%)	29 (39.7%)	0.60
SGLT-2 inhibitor	10 (1.9%)	7 (3.0%)	4 (3.8%)	3 (4.1%)	0.49
Sacubitril-valsartan	12 (2.3%)	8 (3.4%)	5 (4.8%)	3 (4.1%)	0.48
LV geometry/function					
LVEDVi (mL/m^2^)	101.8 ± 39.8	71.3 ± 20.3	61.6 ± 16.4	51.1 ± 18.0	**<0.001**
LV dilation^a^	216 (40.4%)	17 (7.3%)	3 (2.9%)	1 (1.3%)	**<0.001**
LVEDV (mL)	192.5 ± 82.1	132.7 ± 45.7	112.2 ± 36.9	92.9 ± 36.7	**<0.001**
LV mass indexed (g/m^2^)	77.0 ± 27.1	63.7 ± 20.7	57.7 ± 18.6	54.6 ± 19.7	**<0.001**
LVEF (%)	42.6 ± 19.1	59.4 ± 13.0	57.3 ± 13.6	58.7 ± 12.7	**<0.001**
LGE (%)	6.7 ± 10.9	2.0 ± 5.1	2.3 ± 5.3	2.1 ± 3.5	**<0.001**
RV geometry/function					
RVEDVi (mL/m^2^)	96.1 ± 34.6	102.1 ± 30.1	119.6 ± 32.1	170.4 ± 151.6	**<0.001**
RV dilation^b^	125 (23.4%)	63 (26.9%)	59 (56.7%)	62 (81.6%)	**<0.001**
RVEDV (mL)	181.7 ± 73.2	190.1 ± 66.6	218.0 ± 71.2	306.8 ± 265.5	**<0.001**
RVEF (%)	45.2 ± 13.6	49.9 ± 9.7	43.0 ± 10.9	34.2 ± 10.4	**<0.001**
TR severity					
Moderate	475 (88.8%)	194 (82.9%)	59 (56.7%)	34 (44.7%)	**<0.001**
Severe	60 (11.2%)	40 (17.1%)	45 (43.3%)	42 (55.3%)	**<0.001**
Regurgitant volume (mL)	29 ± 9	32 ± 14	35 ± 10	43 ± 20	**<0.001**
Regurgitant fraction (%)	46 ± 12	46 ± 12	41 ± 9	42 ± 11	0.063

Values are mean ± SD or count (percentage).

Abbreviations as in Table 1.

a LVEDVi >108 mL/m^2^ for men; LVEDVi >96 mL/m^2^ for^14^ women.

b RVEDVi >109 mL/m^2^ for men; RVEDVi >97 mL/m^2^ for^14^ women.

### Follow-up and survival

**Figure 1:**
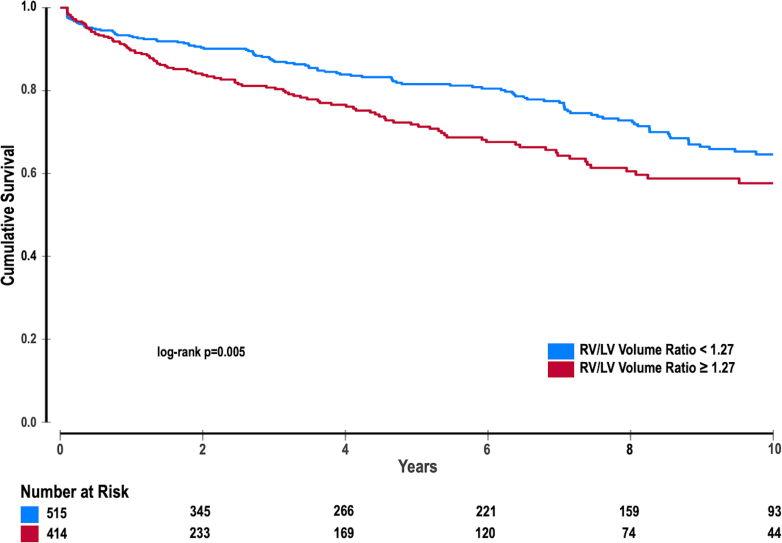
Kaplan-Meier Curve Survival Stratified by Normal and Abnormal Right Ventricular-to-Left Ventricular Volume Ratio LV = left ventricle; RV = right ventricle.

Over a mean follow-up period of 4.8 ± 4.5 years, 236 patients (24.9%) died. Kaplan-Meier mortality estimate was higher among patients with an abnormal RV/LV_vol_ ratio compared to those with a normal ratio (27.7% ± 3.0% vs 19.5% ± 2.0%, log-rank *P* = 0.005). In multivariable Cox regression analyses, adjusting for age, sex, RVEDVi, RVEF, LVEF, LVEDVi, and LGE, the RV/LV_vol_ ratio was a significant independent predictor of all-cause mortality (aHR: 1.47, 95% CI: 1.01-2.14, *P* = 0.043) (Figure 1, Table 3). In a subgroup of patients with TR quantification (n = 453), the RV/LV_vol_ ratio remained a significant predictor of all-cause mortality (aHR: 1.19, 95% CI: 1.04-1.37, *P* = 0.010) after adjusting for the previously noted covariates as well as TR severity itself (Supplemental Table 1).

**Table 3:** Predictors of Mortality

Variable	Univariable Analysis	*P* Value	Multivariable Analysis	*P* Value
	HR (95% CI)		HR (95% CI)	
Age (y)	1.02 (1.01-1.03)	**<0.001**	1.03 (1.02-1.04)	**<0.001**
Male	0.96 (0.74-1.24)	0.79	0.73 (0.55-0.97)	**0.031**
RVEF (%)^a^	0.81 (0.72-0.88)	**<0.001**	0.90 (0.78-1.04)	0.15
RVEDVi (mL/m^2^)^a^	1.06 (1.02-1.09)	**<0.001**	1.06 (1.02-1.11)	**0.007**
LVEF (%)^a^	0.89 (0.83-0.95)	**0.002**	0.89 (0.78.-1.01)	0.081
LVEDVi (mL/m^2^)^a^	1.03 (1.001-1.06)	**0.039**	0.97 (0.92-1.03)	0.46
LGE (%)	1.02 (1.01-1.03)	**0.009**	1.01 (1.00-1.03)	0.089
RV/LV_vol_ ratio (continuous)	1.10 (1.01-1.19)	**0.015**		
RV/LV_vol_ ratio (dichotomous)	1.44 (1.11-1.86)	**0.005**	1.47 (1.01-2.14)	**0.043**
RV/LV_vol_ ratio categories (normal as reference)				
Mild	1.17 (0.86-1.61)	0.300		
Moderate	1.66 (1.10-2.50)	**0.014**		
Severe	2.20 (1.31-3.06)	**<0.001**		

Bold values indicate statistical significance (*P* < 0.05).

RV/LV_vol_ ratio = right ventricular to left ventricular volume ratio; other abbreviations as in Table 1.

a Per 10 unit Δ.

**Figure 2:**
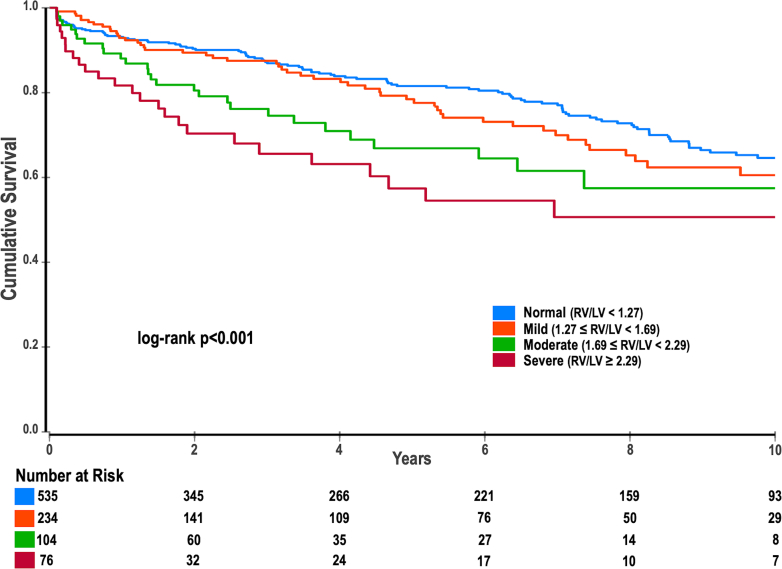
Kaplan-Meier Curve Survival Stratified by the Severity Strata of Right Ventricular-to-Left Ventricular Volume Ratio LV = left ventricle; RV = right ventricle.

The severity strata of the RV/LV_vol_ ratio were strongly associated with all-cause mortality, with mortality rates increasing stepwise from 21.7% ± 3.2% for mild strata to 33.1 ± 6.0% for moderate and 42.6% ± 7.1% for severe (log-rank *P* < 0.001) (Figure 2). In multivariable Cox regression analysis adjusting for age, sex, RVEDVi, RVEF, LVEF, LVEDVi, and LGE, worsening severity strata of the RV/LV_vol_ ratio was a significant independent predictor of all-cause mortality (aHR: 1.31, 95% CI: 1.03-1.66, *P* = 0.024). Compared to the normal RV/LV_vol_ ratio, those in the mild (aHR: 1.47, 95% CI: 0.99-2.19, *P* = 0.054) and moderate (aHR: 1.76, 95% CI: 1.01-3.05, *P* = 0.044) categories were associated with an increased mortality risk, while severe strata carried the highest risk (aHR: 2.20, 95% CI: 1.31-4.76, *P* = 0.045), demonstrating a stepwise increase in risk as the RV/LV_vol_ ratio worsens.

**Figure 3:**
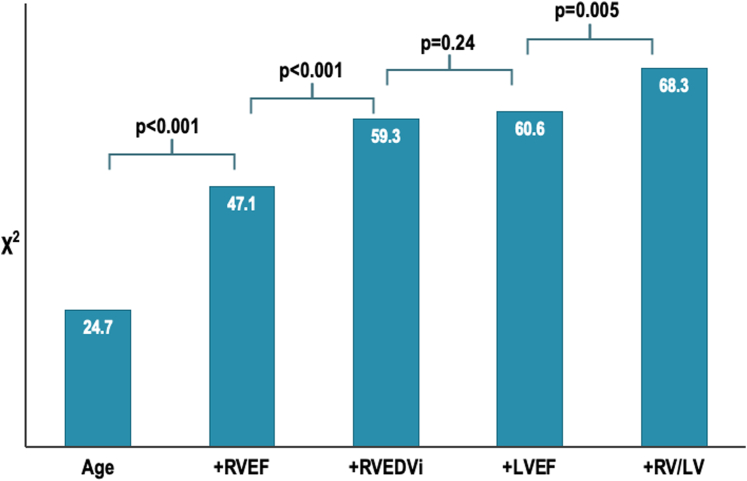
Bar Graph Incremental prognostic value of RV/LV volume ratio over age, RVEF, RVEDVi, and LVEF. LV = left ventricle; LVEF = left ventricular ejection fraction; RV = right ventricle; RVEDVi = right ventricular end-diastolic volume index; RVEF = right ventricular ejection fraction.

The incremental prognostic value of RV/LV_vol_ ratio was further demonstrated through sequential improvements in model performance, as illustrated by rising chi-square values with each added prognostic factor. Baseline models including age yielded a chi-square value of 24.7. The addition of RVEF and RVEDVi improved the model, raising the chi-square to 47.1 (*P* < 0.001) and 59.3 (*P* < 0.001), respectively (Figure 3). There was no statistically significant increase after adding LVEF to the model (chi-square to 60.6 [*P* = 0.24]). Finally, the addition of RV/LV_vol_ ratio, significantly improved model performance with a final chi-square value of 68.3 (*P* = 0.005). These findings were similar when replacing the RV/LV_vol_ ratio as a dichotomous variable (normal/abnormal) with a categorical variable (severity strata), with the model achieving a final chi-square value of 71.5 (*P* = 0.001).

### Survival stratified by TR severity and RV/LV_vol_ ratio

**Figure 4:**
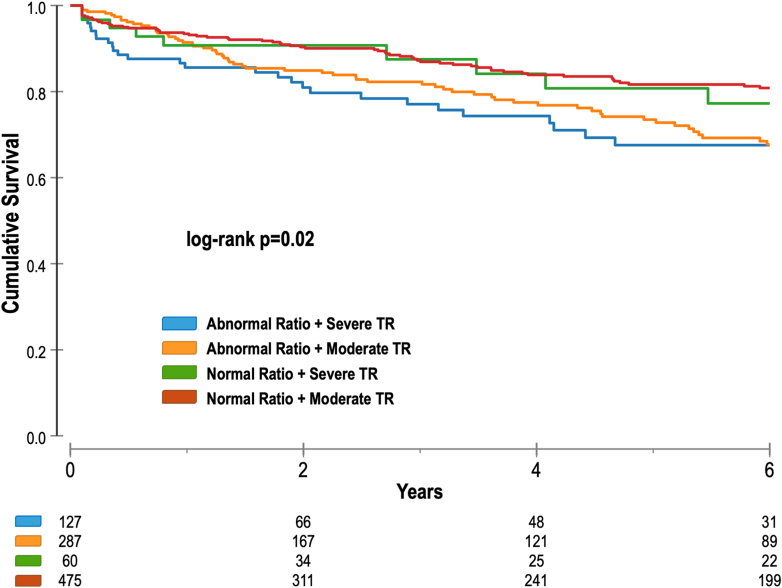
Kaplan-Meier Curve Survival Stratified by the Right Ventricular-to-Left Ventricular Volume Ratio and Tricuspid Regurgitation Severity TR = tricuspid regurgitation.

When stratifying patients by RV/LV_vol_ ratio (normal vs abnormal) and TR severity (moderate vs severe), a stepwise decline in survival was observed across the groups (log-rank *P* = 0.020) (Figure 4). Patients with an abnormal RV/LV_vol_ ratio had worse outcomes compared to those with a normal ratio, with the highest risk seen in those with both severe TR and an abnormal RV/LV_vol_ ratio. Importantly, even among patients with moderate TR, an abnormal RV/LV_vol_ ratio was associated with worse survival, demonstrating its incremental prognostic value beyond TR severity.

### Survival stratified by PH and RV/LV_vol_ ratio

Given the established impact of PH on RV remodeling and outcomes, we performed a stratified Kaplan-Meier survival analysis based on the presence or absence of PH and RV/LV_vol_ ratio category (normal vs abnormal). As shown in Supplemental Figure 1, the prognostic association of the RV/LV_vol_ ratio persisted regardless of PH status, supporting the incremental prognostic value of the RV/LV_vol_ ratio beyond PH status. While patients with both an abnormal ratio and PH had the worst outcomes, the RV/LV_vol_ maintained its prognostic significance in both PH-positive and PH-negative groups.

## Discussion

This study is the first, to our knowledge, to systematically evaluate the RV/LV_vol_ ratio as a marker of ventricular remodeling and outcomes in TR, providing novel insights into its clinical utility. Findings demonstrate that RV/LV_vol_ ratio can detect significant RV remodeling even in patients with normal RVEDVi, with over a third of patients with normal RVEDVi having an abnormal RV/LV_vol_ ratio, and nearly one-fifth of those with severely abnormal RV/LV_vol_ ratio still meeting normal RVEDVi criteria. Additionally, an abnormal RV/LV_vol_ ratio was more prevalent in women and those with lower body surface area, suggesting its particular utility in populations where standard indices may underestimate remodeling due to reliance on absolute thresholds. The RV/LV_vol_ ratio also showed a strong association with stepwise ventricular remodeling, with higher ratios correlating with decreased LVEDVi and LV mass and increased RVEDVi and RV dilation, reflecting progressive disease severity. Importantly, the RV/LV_vol_ ratio was independently associated with increased mortality risk, even after adjusting for conventional risk markers including RVEDVi, RVEF, and TR severity, highlighting its prognostic value as a robust tool for identifying high-risk patients with advanced TR.

Traditionally, RV assessment has relied heavily on RVEDVi, a metric that has several limitations. First, RVEDVi has a broad range of normal values, which means that patients with early or subtle RV enlargement may still fall within the normal range, even though their RV may be dilated compared to their LV.^14^^,^^18^ Furthermore, RVEDVi is influenced by factors such as body surface area, age, and sex, which can complicate its interpretation for individual patients. Lastly, because RVEDVi assesses the RV in isolation and relies on generalized population norms, it does not consider individual variations in LV size and structure, which are essential for a more accurate evaluation of RV size and its impact. The RV/LV_vol_ ratio addresses these limitations by directly measuring the relationship between the ventricles and incorporating both RV dilation and its impact on LV geometry, providing a more comprehensive assessment of TR and its hemodynamic consequences. Our study demonstrates the clinical value of this approach, as the RV/LV_vol_ ratio provided significant incremental prognostic value beyond that offered by RVEF, RVEDVi, and TR severity alone.

In chronic volume overload resulting from TR, the crescent-shaped RV undergoes dilation and becomes increasingly spherical.^19^ With limited pericardial capacity, this triggers a leftward shift of the ventricular septum, altering LV geometry and impairing LV filling.^19^ The resulting reduction in cardiac output, coupled with diastolic dysfunction and elevated left-sided filling pressures, leads to further maladaptive RV remodeling.^20^ This process also displaces the papillary muscles, leading to tricuspid valve leaflet tethering and perpetuating a vicious cycle where TR begets TR. The RV/LV_vol_ ratio serves as a marker of this ventricular interdependence, a key mechanism in the pathophysiology of RV failure. Interestingly, in our study, patients with elevated RV/LV_vol_ ratio had higher rates of mortality despite having higher LVEF and lower scar burden compared to those with a normal RV/LV_vol_ ratio. This paradoxical finding challenges the traditional notion that reduced LVEF is linked to poor outcomes in advanced TR.^21^ This counterintuitive observation highlights the complex interplay between RV and LV function. Consistent with this, we observed that patients with abnormal RV/LV_vol_ ratio had lower LVEDVi, with those in the severely abnormal category showing the smallest LVEDVi, the largest RVEDVi, and the lowest RVEF. These findings provide a physiological basis for the prognostic importance of the RV/LV_vol_ ratio, emphasizing its role in capturing right and left heart interactions.

Finally, our study also highlights the clinical relevance of the RV/LV_vol_ ratio in the context of evolving management strategies for TR. Historically regarded as the “forgotten valve,” management of TR has largely focused on medical therapy, which has shown limited efficacy in improving mortality. Surgical intervention, historically associated with high morbidity and mortality rates, has often been underutilized and may not necessarily offer advantages over medical therapy.^22^^,^^23^ However, the emergence of TVI has created a need for more refined and objective metrics to guide intervention timing. Based on current guidelines, the only class I indication for tricuspid valve surgery is in patients with severe TR undergoing left-sided valve surgery (class IB-NR).^2^ In asymptomatic patients, the indication for surgical intervention is progressive RV dysfunction.^2^ Therefore, assessment of RV dimensions and function is crucial to select patients who may benefit from TVI.

The RV/LV_vol_ ratio, by reflecting both RV and LV interactions, could serve as a critical parameter in identifying patients who would benefit most from early intervention. Additionally, the study’s findings align with prior research demonstrating the prognostic significance of the RV/LV_vol_ ratio in pulmonary hypertension and pulmonic regurgitation,^4^, ^5^, ^6^^,^^24^ further validating its role as a marker of adverse outcomes in diseases affecting the right heart. Its clinical utility is further strengthened by its ease of use, as it can be derived directly from standard CMR volumetric imaging without the need for complex calculations or advanced magnetic resonance imaging technology. This combination of prognostic value and simplicity makes the RV/LV_vol_ ratio a practical and effective tool in routine clinical practice.

### Study Limitations

This study has several limitations that should be acknowledged. First, as a single-center study, its findings may not be generalizable to broader populations, and the retrospective design inherently introduces potential biases. Second, while the RV/LV_vol_ ratio was evaluated using CMR, the gold standard for volumetric assessment, the accessibility of CMR may limit its widespread clinical application. Third, while the study focused on all-cause mortality, investigating the relationship between RV/LV_vol_ ratio and other clinical outcomes, such as heart failure hospitalizations, or quality of life, should also be explored. Fourth, PH was the underlying etiology of functional TR in approximately one-third of patients. In this setting, progressive RV dysfunction and uncoupling from the pulmonary circulation are known to drive adverse outcomes. Right ventricular-pulmonary arterial uncoupling, which may be assessed by surrogate markers such as RVEF or tricuspid annular plane systolic excursion/pulmonary artery systolic pressure ratio, reflects the inability of the RV to adapt to increased afterload. In our study, RVEF was a strong predictor of mortality in univariable analysis and showed borderline significance in multivariable models after adjustment for other CMR-derived indices, including RV/LV_vol_ ratio. This may suggest that the RV/LV_vol_ ratio serves as a surrogate marker of right ventricular-pulmonary arterial uncoupling; however, our study did not directly evaluate this relationship due to the absence of echocardiographic or invasive hemodynamic data. Further studies are warranted to explore this potential association.

## Conclusions

This study establishes the RV/LV_vol_ ratio as a robust predictor of mortality in TR, emphasizing its potential to augment current risk stratification methods and guide therapeutic decision-making. Its incorporation into clinical practice, particularly in the era of expanding transcatheter therapies, could help optimize outcomes for patients with TR. Further research is warranted to validate these findings across other populations and explore its role in predicting outcomes beyond mortality.

Perspectives **COMPETENCY IN PATIENT CARE:** This study highlights the clinical significance of the RV/LV_vol_ ratio in advanced TR, providing incremental value beyond traditional metrics including RVEDVi, RVEF, and TR severity. By capturing the interactions between the RV and LV, the RV/LV_vol_ ratio provides a window into ventricular interdependence and its implications in clinical outcomes. Integrating RV/LV_vol_ ratio into clinical assessment has the potential to refine patient risk stratification, inform therapeutic decision-making, and ultimately improve patient care in advanced TR.

**TRANSLATIONAL OUTLOOK:** Future efforts should focus on integrating RV/LV_vol_ ratio into risk stratification algorithms to guide therapeutic decision-making, particularly in identifying candidates who may benefit from early transcatheter tricuspid interventions. Multidisciplinary collaboration between imaging specialists, heart failure clinicians, and interventional cardiologists will be critical to validating and incorporating this metric in prospective studies and clinical pathways.

## Funding support and author disclosures

This study was funded by the 10.13039/100000050National Institutes of Health/National Heart, Lung, and Blood NIH/NHLBI R01HL159055 (to Dr Kim) and NIH/NHLBI R01HL170566 (to Drs Kim and Weinsaft). All other authors have reported that they have no relationships relevant to the contents of this paper to disclose.
