# Supplementary material for: Prognostic Value of the Right Ventricular-to-Left Ventricular Volume Ratio in Tricuspid Regurgitation
Source: JACC Adv. 2025 Jul 4;4(8):101922. doi: 10.1016/j.jacadv.2025.101922 (PMC12271060; doi:10.1016/j.jacadv.2025.101922)

**Supplemental Table 1**: Univariable and Multivariable Cox Regression Analysis for Mortality in Subgroup Analysis (n=453)

| **Variable** | **Univariable Analysis** | **p** | **Multivariable Analysis** | **p** |
| --- | --- | --- | --- | --- |
|  | **Hazard Ratio (95% CI)** |  | **Hazard Ratio (95% CI)** |  |
| Age (years) | 1.03 (1.01-1.04) | **< 0.001** | 1.03 (1.02-1.05) | **<0.001** |
| Male | 0.82 (0.58-1.17) | 0.28 | 0.82 (0.56-1.21) | 0.32 |
| TR regurgitant fraction strata^†^ | 1.45 (1.05-2.01) | **0.023** | 1.22 (0.85-1.74) | 0.27 |
| RVEF (%)* | 0.83 (0.72-0.96) | **0.013** | 0.84 (0.71-1.01) | 0.061 |
| RVEDVi (ml/m^2^)* | 1.02 (0.98-1.06) | 0.29 | 1.00 (0.95-1.06) | 0.83 |
| LGE (% LV) | 1.02 (1.00-1.04) | 0.07 | 1.03 (1.01-1.05) | **0.005** |
| RV/LV_vol_ ratio (continuous) | 1.20 (1.07-1.33) | **0.001** | 1.19 (1.04-1.37) | **0.010** |

*per 10 unit Δ

†TR regurgitant fraction: <30%, 30-49%, ≥50%^11^

CI, confidence interval; HR, hazard ratio; LGE, late gadolinium enhancement, LVEF, left ventricular ejection fraction; RVEDVi, right ventricular end-diastolic volume index; RVEF, right ventricular ejection fraction; RV/LV_vol_ ratio, right ventricular to left ventricular volume ratio.

1A: Intra-rater and inter-rater reliability of tricuspid regurgitant volume and fraction In 20 patients, tricuspid regurgitant volume and fraction were independently assessed by two blinded observers for inter-rater reliability and one observer for intra-rater reliability. The intraclass correlation coefficient (ICC) for TR regurgitant volume was 0.90 (95% CI: 0.77–0.96, p<0.001) for inter-rater reliability and 0.91 (95% CI: 0.81–0.97, p<0.001) for intra-rater reliability, while the ICC for TR regurgitant fraction was 0.86 (95% CI: 0.68–0.94, p<0.001) and 0.89 (95% CI: 0.75–0.95, p<0.001), respectively.

**Supplemental Figure1:** Kaplan-Meier survival curves stratified by pulmonary hypertension (PH) and RV/LV volume ratio.


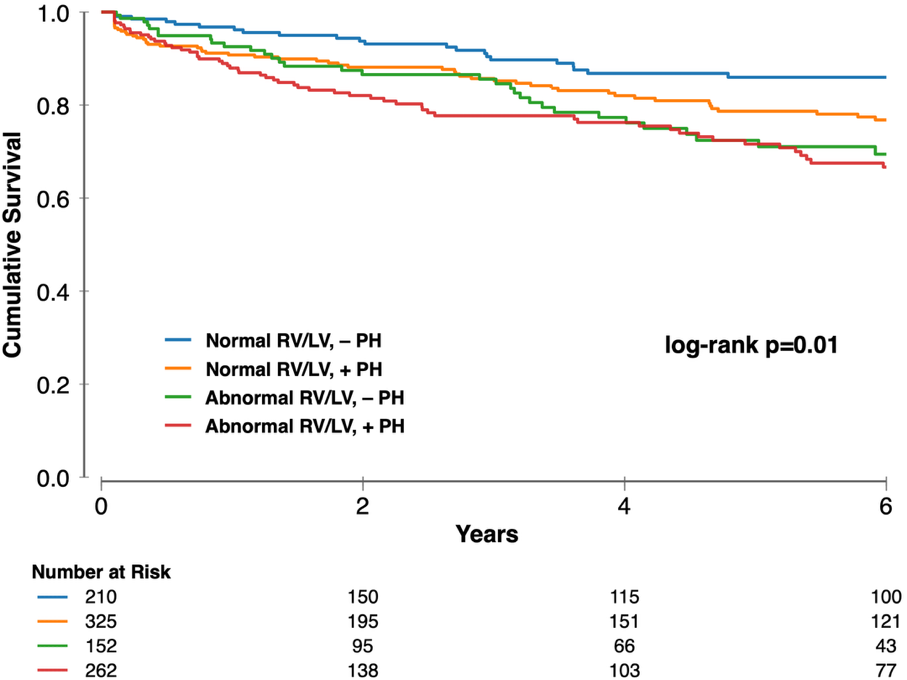

Supplement: Supplemental Material [file mmc1.docx]
